# Single-step-fabricated disordered metasurfaces for enhanced light extraction from LEDs

**DOI:** 10.1038/s41377-021-00621-7

**Published:** 2021-09-06

**Authors:** Peng Mao, Changxu Liu, Xiyan Li, Mengxia Liu, Qiang Chen, Min Han, Stefan A. Maier, Edward H. Sargent, Shuang Zhang

**Affiliations:** 1 School of Physics and Astronomy, University of Birmingham, B15 2TT Birmingham, UK; 2 National Laboratory of Solid State Microstructures, College of Engineering and Applied Sciencesand Collaborative Innovation Centre of Advanced Microstructures, Nanjing University, Nanjing, 210093 China; 3 Chair in Hybrid Nanosystems, Nanoinstitute Munich, Faculty of Physics, Ludwig-Maximilians-Universitaet Muenchen, 80539 Muenchen, Germany; 4 Department of Electrical and Computer Engineering, University of Toronto, 35 St. George Street, Toronto, ON M5S 1A4 Canada; 5 Key Laboratory of Intelligent Optical Sensing and Manipulation (Nanjing University), Ministry of Education, Nanjing, China; 6 Department of Physics, Imperial College London, London, SW7 2AZ UK; 7 Department of Physics, University of Hong Kong, Hong Kong, 999077 China; 8 Department of Electrical and Electronic Engineering, University of Hong Kong, Hong Kong, 999077 China

**Keywords:** Inorganic LEDs, Nanoparticles

## Abstract

While total internal reflection (TIR) lays the foundation for many important applications, foremost fibre optics that revolutionised information technologies, it is undesirable in some other applications such as light-emitting diodes (LEDs), which are a backbone for energy-efficient light sources. In the case of LEDs, TIR prevents photons from escaping the constituent high-index materials. Advances in material science have led to good efficiencies in generating photons from electron–hole pairs, making light extraction the bottleneck of the overall efficiency of LEDs. In recent years, the extraction efficiency has been improved, using nanostructures at the semiconductor/air interface that outcouple trapped photons to the outside continuum. However, the design of geometrical features for light extraction with sizes comparable to or smaller than the optical wavelength always requires sophisticated and time-consuming fabrication, which causes a gap between lab demonstration and industrial-level applications. Inspired by lightning bugs, we propose and realise a disordered metasurface for light extraction throughout the visible spectrum, achieved with single-step fabrication. By applying such a cost-effective light extraction layer, we improve the external quantum efficiency by a factor of 1.65 for commercialised GaN LEDs, demonstrating a substantial potential for global energy-saving and sustainability.

## Introduction

Lighting accounts for 15% of global electricity consumption and 5% of worldwide greenhouse gas emissions^1^. Achieving energy-efficient light sources is hence of utmost importance for global development and sustainability. By virtue of its compact size, high efficiency and long lifetime, the light-emitting diode (LED) has developed into the prime candidate for replacing conventional light sources such as incandescent bulbs and fluorescent tubes. However, owing to high costs, less than 10% of existing lighting installations use LED products in most regions of the world, even with government policy support^2^. A 50% rise in lighting demand by 2030 is predicted due to population growth and increased urbanisation^1^. Consequently, improving the efficiency of LED sources while minimising the cost becomes an urgent challenge for both science and engineering communities.

With substantial efforts in the past decades, prominent progress has been made in LED materials quality, particularly of both III-Nitrides and organics, leading to an internal quantum efficiency close to unity^3,4^. However, a considerable fraction of generated photons is trapped inside the high-index materials of LEDs by total internal reflection (TIR), rendering outcoupling efficiency the bottleneck of overall efficiency and hence energy saving. Methods have been developed to extract light from highly confined waveguide modes, including fabrication of dielectric/metallic nanostructures on the top surface or the substrate^5–23^, patterned electrodes^24–26^ and corrugations inside the bulk^27–32^. Despite reported output power improvement of more than two times compared to unstructured devices^6,8–11,18,24,27,28^, light-extraction structures always require sophisticated and time-consuming fabrication processes, including template fabrication, lithography (by optics or electron beam) or nanoimprint. From a practical point of view, the added fabrication cost of the structures can outweigh the benefit of enhancement of the external quantum efficiency, hindering their practical application at the industrial level.

Lithography-free structuring of LED substrates^7,12,22,23^ had been proposed to ease the fabrication and integration. However, multiple-step processes are still needed; and the nanostructures are usually fabricated underneath the active region, which make the devices vulnerable to electrical failure due to highly localised electric fields or shorts. There is a trade-off between efficiency and fabrication complexity—simplified fabrication is reached at the cost of reduced efficiency enhancement (See more details in Supplementary Note 1). Last but not least, lithography-free solutions are usually not compatible with mainstream III–V solid-state technology. To this end, a cost-effective solution that can be applied to commercialised LED systems remains elusive.

**Fig. 1:**
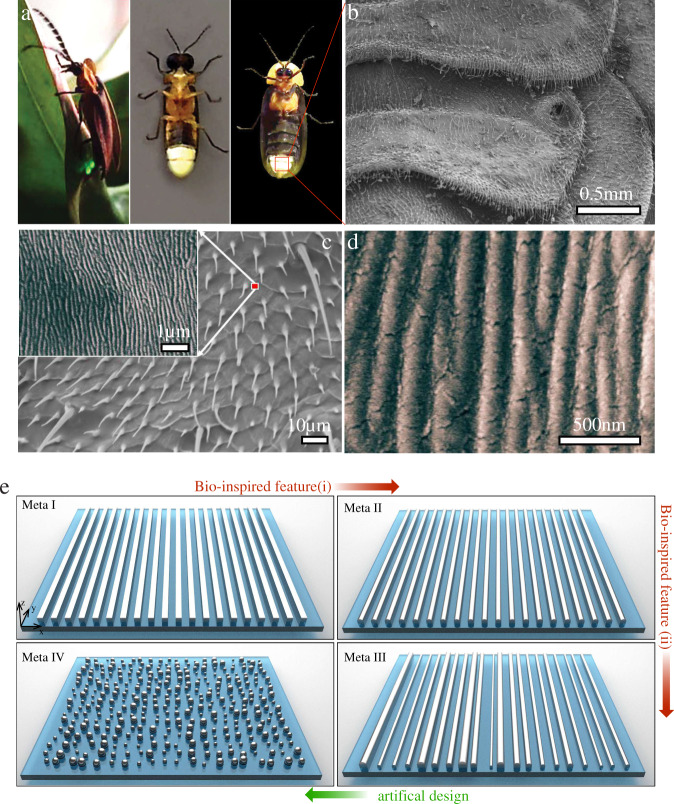
Evolution-inspired design of disordered metasurface for light extraction. **a** Photo images of Photinus, Phausis reticulata and Pyrocoelia rufa. **b** The SEM image of lantern cuticles of Pyrocoelia rufa. **c** Enlarged view of microstructures on the lantern cuticle of the firefly. The inset shows the microstructures with higher magnification. The disorder in both position and size of stripes can be observed. **d** High magnification SEM image of microstructure on the lantern cuticle. The curvature on the top surface for each stripe is clearly demonstrated. **e** The design flow of the metasurface. Meta-I is an ordinary metasurface composed of periodic stripes with squared cross-sections. Meta-I evolves to Meta-II and Meta-III by utilising two bio-inspired features, the curved top surfaces and disorder, respectively. Meta-IV endows extend features to another dimension, transforming stripes to nanoparticles

Evolution-driven photonic structures may provide new solutions to improve the extraction efficiency of LEDs. Nature developed optimised energy harvesting and management systems through millions of years of evolution. Evolution-driven photonic structures have provided intriguing clues to improve the performance of optical systems, ranging from passive cooling by Saharan silver ants^33^ to broadband solar absorbers by white beetles^34^, black butterflies^35^ or viola flowers^36^. Here, we draw inspiration from fireflies (as shown in Fig. 1a) to the photon management of LEDs, achieving a simultaneous high efficiency and ease of fabrication. Based on a metasurface^37,38^ composed of disordered silver nanoparticles (NPs) on top of unstructured GaN LEDs, we improve the output power by 2.7 times for as-prepared commercialised devices in photoluminescence measurement. After packaging with semi-spherical epoxy, a prominent improvement of absolute external quantum efficiency (EQE) is achieved (from 31% to 51%). Most importantly, we realise this light extraction layer by a single procedure based on gas-phase cluster beam deposition^39^, drastically reducing fabrication cost and time. In conjunction with scalability to large-area manufacturing, our cost-effective disordered metasurfaces represent a promising approach for bridging the gap between high-efficient laboratory demonstration and industrial production in the lighting market.

## Results

**Fig. 2:**
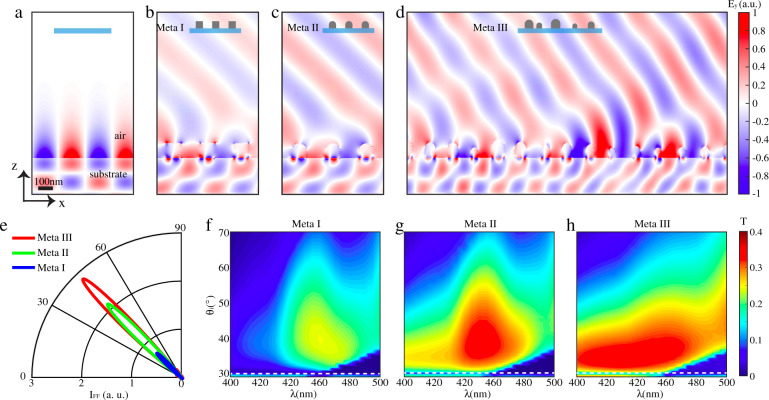
FDTD simulations for the evolution-inspired light extraction. **a** The field distribution *E*_*y*_ of a substrate without any light extracting structures. When the incident light is beyond critical angle, no photons can escape. **b**–**d** The field distribution *E*_*y*_ of a substrate with (**b**) Meta-I, (**c**) Meta-II and (**d**) Meta-III. The amplitude of the electric field coupled outside is gradually increased, demonstrating the improvement of light extraction by utilising the bio-inspired features. **e**, A far-field intensity for three different metasurfaces. For simulations in (**a**–**d**), the incident angle is chosen as *θ*_*i*_ = 32^o^ while the critical angle for TIR is *θ*_*c*_ = 30^o^. The wavelength of the light is at 450 nm. **f**–**h** The transition spectra of the substrate at different incident angles beyond *θ*_*c*_; (**f**) Meta-I, (**g**) Meta-II and (**h**) Meta-III. For disordered Meta-III, 3 samples with different sets of random variables are investigated. The white dashed line corresponds to *θ*_*c*_. A prominent improvement is observed for different angles and wavelength from Meta-I to Meta-II. The region with efficient light extraction (reddish colour) is enlarged by the introduction of disorder (Meta-III)

We develop our light extraction structure by drawing on light manipulation techniques via metasurfaces and geometric abstraction from fireflies, taking advantages from both artificial design and natural selection. Figure 1 summarises the design procedure assisted by bio-inspiration and Fig. 2 illustrates the detailed numerical analysis, which elucidates the exceptional power of this evolution-inspired approach.

We start optimising the light extraction layer based on a metasurface composed of periodic metallic stripes with a square cross-section (Meta-I), as depicted in the top-left panel of Fig. 1e. We choose the material of the metasurface to be silver, one of the most commonly used noble metals due to its strong plasmonic response. The size of the stripe cross-section is 100 nm and the periodicity is 230 nm. To demonstrate the light extraction effect of Meta-I, we first take a dielectric substrate with refractive index *n* = 2 as a reference, which exhibits a critical angle $\theta _c = \arcsin \frac{1}{n} = 30^o$ for light emitted from the substrate into air. In the absence of metasurfaces, the electric field decreases exponentially away from the interface for incident angle $\theta _c = 32^o$, which is greater than *θ*_*c*_. The corresponding spatial distribution of the electric field component (*E*_*y*_) is illustrated in Fig. 2a, implying no photons are able to escape the substrate. The direction of y is shown in Fig. 1e and the wavelength of the incident light is selected as 450 nm. After the introduction of the metasurface top layer consisting of a periodic array of silver stripes with a rectangular cross-section, new outcoupling channels are created through excitation of surface plasmon via wavevector compensation from the periodicity of the structure^40–42^, releasing a portion of otherwise trapped photons into air. Figure 2b explicitly demonstrates this effect of photon extraction.

To lift the limitation of extraction ability of a standard structure, we sought insight from biological light extraction systems, which have obtained highly optimised features through evolution. In particular, we investigate three types of fireflies (Pyrocoelia rufa, Photinus and Phausis reticulata) living in east Asia with green or yellow light luminescence, as shown in Fig. 1a. Interestingly, the morphologies of the abdominal segment of all three fireflies are similar, implying some generic mechanism for efficient light extraction. The SEM images of the abdominal segment of Pyrocoelia rufa are illustrated in Fig. 1b–d, while SEM images of the other two can be found in Supplementary Note 2. The geometry of the bioluminescence organ provides some clues for a better light-extraction solution. We abstract two geometric features that are optimised for light extraction: (i) the curved top for each stripe (Fig. 1d); and (ii) the global disorder in both location and size of the stripes (Fig. 1c).

By incorporating feature (i) into the original periodic metasurface Meta-I, we obtain a new nanostructure Meta-II, which is depicted in the top-right panel of Fig. 1e. The intensity (square of peak values of the electric field as shown in Fig. 2c) is enhanced compared to the ones shown in Fig. 2b), demonstrating an improved outcoupling by the curved top. Based on the feature (ii), randomness in both size and position is introduced for each unit, resulting in a disordered metasurface Meta-III, as shown in the bottom-right panel of Fig. 1e. Detailed geometric parameters can be found in the Methods section. A higher transmission compared to the periodic cases is evident from the spatial distribution shown in Fig. 2d. To provide a quantitative comparison, we calculate the far-field intensity I_FF_ of the different metasurfaces, as demonstrated in Fig. 2e. The increased far-field intensity elucidates the power of the two bio-inspired features for improving light extraction beyond TIR.

We further investigate the wavelength dependence of transmission T for the three different metasurfaces, as summarised in Fig. 2f–h. The transmission spectra T are calculated at different incident angles beyond the critical angle (*θ*_*c*_ > *θ*_*i*_), to provide a comprehensive analysis. A prominent transmission improvement is achieved by optimising the top half of the element from a flat (Fig. 2f) to a curvilinear surface (Fig. 2g). After the introduction of disorder, the maximum value of T remains the same level. However, efficient light extraction (high transmission) can be achieved in a broader spectral region (Fig. 2h), taking advantage of randomised wavevector compensation. A broadband transmission is observed far beyond the TIR condition in this case. A portion of photons can be extracted even with an incident angle beyond 60^o^. More details about simulations can be found in the Methods section. A detailed investigation of the relationship between extraction efficiency and the level of disorder is shown in Supplementary Note 3.

Instead of utilising the bio-inspired structure directly, we implement an additional optimisation step, acquiring Meta-IV as depicted in the lower-left panel of Fig. 1e. We extend both the disorder and curved surface to a second dimension, obtaining a disordered metasurface with randomly allocated NPs with curved top surfaces but flat bottom surface. Besides the enhancement of light extraction summarised in Fig. 2, Meta-IV acquires two additional advantages. Firstly, the reduced top cover ratio increases the photon transmission for optical rays inside the light cone (more details can be found in Supplementary Note 4). More importantly, such a NP-based metasurface provides the possibility for single-step fabrication, a crucial point for cost-effective devices.

**Fig. 3:**
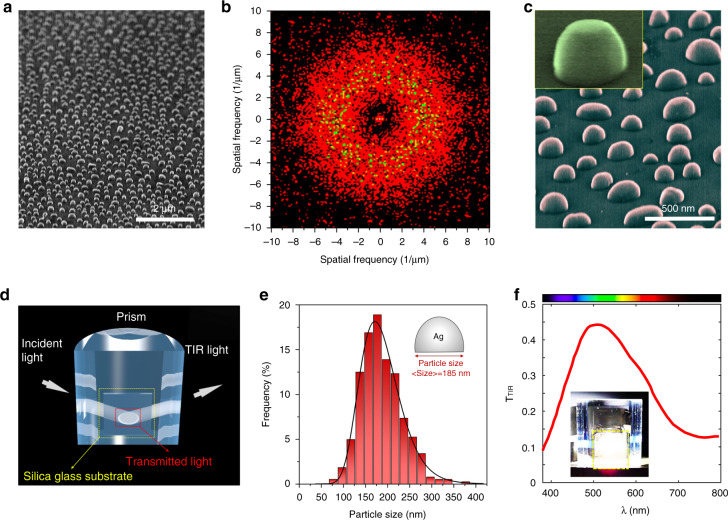
Characterisations of the fabricated disordered metasurface Meat-IV. **a** Low magnification SEM image of the disordered Ag metasurface. **b** Two-dimensional Fourier power spectrum of the position of the Ag nanostructures, which is calculated from the SEM image of disordered Ag metasurface. **c** The tilted view of the disordered metasurfaces with high magnification. Inset is image of a single Ag nanostructure. **d** A schematic illustration of the light extraction measurement setup. **e** Size distribution histogram of the Ag nanoparticles in the optimised disordered metasurface and corresponding Log-Normal fitting (black solid line). **f** Transmission spectrum from the hemispherical glass prisms with Meta-IV for *θ*_*i*_ = 50^o^. The SiO_2_ substrate covered with disordered Ag metasurface and the prism were bonded together by using index-matching fluid (*n* = 1.46). Inset: optical image of the spot of transmitted light extracted from the glass prism with disordered Ag metasurface

We leverage the gas-phase cluster beam technique to fabricate the designed structure Meta-IV, exploiting its capability to form metallic NPs by a single non-intrusive deposition step. More details of the fabrication can be found in the Methods section. Figure 3a shows a scanning electron microscope (SEM) image of a representative disordered metasurface, illustrating a qualitatively good match to the original design in Fig. 1e. Disorder is intrinsically ensured during the process by randomly allocating NPs with different sizes. For a quantitative illustration, we performed a two-dimensional Fast Fourier Transform (2D-FFT) based on the positions of the Ag NPs. A circle-shaped distribution of the squared Fourier components is observed in Fig. 3b, confirming the disordered arrangement of Ag NPs^43^. Figure 3c provides a tilted view of the disordered metasurfaces under high magnification. The two critical geometric features, the disorder (in both size and position) and the curved top, are clearly demonstrated.

Besides single-step fabrication, the gas-phase cluster beam technique offers the flexibility to tune the size and density of NPs statistically via controlling deposition time and temperature. In experimental realisations, disorder in position cannot be controlled. Therefore, instead of 3D simulations that require formidable computational resources, we directly optimise the Meta-IV structure experimentally for the best light extraction ability. More details are in Supplementary Note 5. Figure 3d depicts the setup for the measurement of the transmission beyond TIR. A broadband light source with incident angle beyond *θ*_*c*_ impinges on a hemicylindrical silica prism coated with our metasurface, with the transmitted light being collected from the other side. Figure3e shows the size distribution of an optimised sample. A Gaussian distribution is achieved with an average value of 185 nm and a standard deviation of 40 nm. The corresponding transmission spectrum T_TIR_ beyond *θ*_*c*_ is shown in Fig. 3f, demonstrating a significant enhancement of light extraction under the TIR condition. With the aid of our disordered metasurface, the otherwise trapped photons can be coupled to the outside continuum, which can be readily distinguished by the naked eye (as shown in the inset of Fig. 3f). The metasurface results in a transmission enhancement of 30% within a broad spectral range (from 440 to 620 nm), covering most visible colours from red to blue. The corresponding colours are demonstrated in the colour bar based on the 1964 CIE 10-degree observer. The value of transmission is better than the simulation results shown in Fig. 2, owing to the structure being extended in a second dimension (from stripes to particles) and more complicated geometry (higher level of disorder) in realistic structures. A comparison with simulated results in 3D can be found in Supplementary Note 6. The angle-dependent experiments are implemented and shown in Supplementary Note 7.

**Fig. 4:**
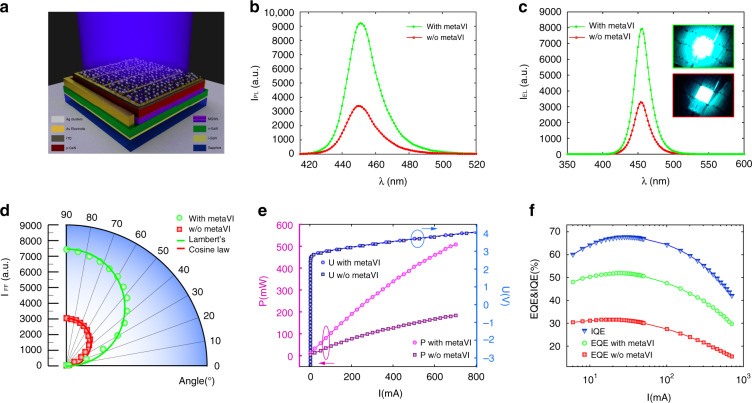
Performance of commercialised LED with disordered metasurface Meat-IV. **a** Schematic illustration (not to scale) of a GaN-based LEDs with disordered metasurface deposited on the top. **b** Photoluminescence spectra of LEDs with/without Meat-IV, with an enhancement of 170%. **c** Electroluminescence spectra of LEDs with/without Meat-IV, with an enhancement of 140% **d** Polar plot for the far-field light intensity of LEDs with/without Meat-IV. The data is fitted with Lambert’s cosine law for each case. **e** The voltage and output power as a function of current for LEDs with/without Meat-IV. The metasurface has negligible impact on light generation of the LED beneath (from I-U) but prominent impact on light extraction (from I-P). **f** IQE and EQE of LEDs with/without Meat-IV. After packaging, the EQE is increased from 31.6% to 51.5% by aid of Meat-1V

To demonstrate the enhancement of outcoupling for a commercially available structure, we incorporate our metasurface into factory-made GaN LEDs. A blue LED is selected for its universal application, not only as one of the three primary colours but also for white light generation with a phosphor coating^44^. To demonstrate the immediate application prospect of our approach, we used commercialised LEDs fabricated by industrial-level technologies (more details in the Methods section). The metasurface is fabricated on the top face of the GaN-LED wafer above the ITO/electrode with a single-step procedure, as depicted in Fig. 4a.

Figure 4b–f summarises the performance of the LEDs with evolution-inspired nanostructures. Identical LEDs without top metasurface are used as a reference. Photoluminescence (PL) and electroluminescence (EL) spectra are shown in Fig. 4b, c, respectively. For the PL, we observe a pronounced emission enhancement of 170% without spectral distortion by utilising Meta-IV. PL intensities at different pump powers can be found in Supplementary Note 8. When the optical pump is replaced by an injection current of 350 mA, a prominent intensity enhancement of 140% persists without notable peak wavelength shift (at 452 nm) nor broadening of the peak width (25 nm), owing to the negligible impact of the top metasurface to the electronic component beneath. Figure 4d compares the far-field radiation profiles I_FF_ of the two types of LEDs, wherein the direction perpendicular to the surface of the LED is defined as 90 degrees. Compared with the control sample, the far-field intensity of LEDs with our disordered metasurface exhibits a noticeable enhancement in a wide angular range from 10^o^ to 90^o^. Benefiting from the metasurface with ultra-thin thickness and disorder, a homogeneous enhancement of emission obeying Lambertian cosine law across the whole angular range is achieved, as a beneficial factor in the lighting application.

To complete the investigation, we characterise the output power (*P*) and voltage (*U*) for different injection currents (*I*), as summarised in Fig. 4e. Interestingly, the difference of the *I*–*U* curves between the LED with metasurface and the control is negligible. We attribute this to the negligible influence from the metasurface to carrier transport in the device, a critical feature that is favourable for light extracting structures. An output power enhancement of 165 ± 21% is reached at different pump currents from 1 mA up to 800 mA. Finally, to demonstrate the applicability of our light extraction metasurface for practical devices, we packaged the LED with semi-spherical epoxy using standard technology and measured the LED efficiency, as shown in Fig. 4f. While sharing the same internal quantum efficiency (IQE), the external quantum efficiency (EQE) of the LED with metasurface exhibits a remarkable boost. By measuring 75 independent devices, we observe an overall EQE improvement from 31.6(±2.6)% to 51.5(±3.8)% by introducing disordered metasurfaces. More details can be found in Supplementary Note 9, 10. The epoxy can protect the Ag nanoclusters from environmental degradation, improving the stability of the whole system. Also, the hemispherical epoxy with a refractive index between that of air and GaN improves light extraction, resulting in a relatively smaller enhancement of EQE in comparison to EL.

## Discussion

Haitz’s law^45^, the LED counterpart of Moore’s law, forecasts that every decade the amount of light generated by an LED increases by a factor of 20, while the cost falls by a factor of 10. We believe that our single-step fabricated, high-efficient light extraction metasurface contributes to the continuous fulfilment of this trend. Instead of directly mimicking bio-structures^17^, we utilise a plasmonic material (Ag) with strong light–matter interaction and implement artificial design for both better performance and simpler fabrication. Compared to other nanoparticle-based^13,16,21^ and random-media-based^5,11,22,28^ structures, not only does our structure achieve a much better light extraction; more importantly, it offers a non-intrusive single-step fabrication with potential of direct commercialisation. By virtue of the compatibility of gas-phase cluster beam deposition, our disordered metasurface can be readily developed on ITO or dielectric substrate with little adverse influence on the device. Based on the transmission spectrum under the TIR condition shown in Fig. 3e, our disordered metasurface could be used for LEDs operating in other visible wavelengths, with comparable or even better performance (considering that the transmission spectrum peak around 500 nm). Moreover, similar strategies may be applied to the next generation of LEDs based on quantum dots or perovskites^46,47^.

Recently, chemically synthesised Ag nanocubes were utilised as light extraction structures for OLED^23^. Despite the enhancement of both efficiency and stability, the effect of the intrinsically endowed disorder of the nanostructures was not investigated. Here, we not only demonstrate a method to fabricate the plasmonic structures with a single step, but we also provide a comprehensive study of the role of disorder played in broadband light extraction. Besides, the enhancement arising from top surface engineering of nanoparticles (from flat to curved one, as shown in Fig. 2) may inspire the further improvement of related devices.

Considering the Helmholtz reciprocity of light, the bio-inspired structure can also be used to effectively couple light into the substrate in the visible region. Not confined to light-extraction purposes, disordered metasurfaces may play a role as an auxiliary layer for absorption enhancement of photovoltaic devices or photodetectors.

## Materials and methods

### Numerical simulations for light extraction structures

All the FDTD simulations are based on a commercialised software (LUMERICAL, FDTD Solution). The refractive index is selected as *n* = 2. The refractive index of Ag is based on the data from ref. ^48^. A plane wave is launched with incident angle *θ*_*i*_ = 32°, i.e., 2 degrees above the critical angle of TIR. The polarisation of the field is along the *x*-axis. The wavelength is selected at λ = 450 nm. Since the length of stripe is much larger than the size of cross-section (as shown in Fig. 1a), a 2D simulation is conducted. This size of the cross-section *d*_0_ is 100 nm and the periodicity of the stripe T is 230 nm. The disorder is introduced to the system by the fluctuations of the size *d*^i^ and position *x*^*i*^ for each element:

1 $$d^i = d_0^i + \Delta _{{\rm{siz}}}U_1\left( {x,y} \right)$$

2 $$x^i = x_0^i + \Delta _{{\rm{pos}}}U_2\left( {x,y} \right)$$

with $x_0^i$the position of the stripes in the periodic array, *d*_0_ the original diameter, *U*_1_ and *U*_2_ independent uniform distributions in [−1 1]. The disorder in position *Δ*_pos_ is 20 nm while in size *Δ*_siz_ is 40 nm. Here, we utilise a plane wave to demonstrate the physical mechanism for the light extraction, especially in the condition of TIR.

### Fabrication and characterisation of Meta-IV

Meta-IV was prepared by gas-phase cluster beam deposition method. The nanocluster beam deposition system is composed of a cluster source, differential vacuum component, particle control component and a deposition chamber. In this fabrication, Ag clusters were generated in a magnetron plasma gas aggregation cluster source. The nanocluster beam was formed by differential pumping induced expansion, and then deposited onto the surface of the LED chips directly. The deposition was performed in a high-vacuum chamber equipped with the cluster source. An Ag target with high purity (99.999%) was used as the sputtering target. The magnetron discharge was operated in an argon stream at a pressure of about 90 Pa in a liquid nitrogen cooled aggregation tube. Ag atoms were sputtered out from the target and Ag NPs were formed through the gas aggregation process in the argon gas. The clusters were swept by the gas stream into high vacuum through a nozzle and a skimmer, respectively, forming a collimated cluster beam with ultrasonic speed. More details in Supplementary Note 5. The operation temperature is 350 ^o^C and the maximum size of the substrate is 15 cm × 15 cm for our home-made setup. The structural properties of the metasurfaces were characterised by scanning electron microscopy (SEM, HITACHI S4500).

The light-transmission properties of the Ag metasurfaces were inspected on a spectrophotometer containing a hemicylindrical prism sample stage. The samples were directly adhered to the flat face of the hemicylindrical silica glass prism. The gap between the sample substrate and the prism was filled with refractive index-matching liquid of refractive index *n* = 1.46. An integrating sphere was placed behind the prism to collect the transmitted light. A white light beam (Ocean Optics, DH2000) passed through the circular edge of the prism and impinged on the centre of the flat face of the hemicylindrical prism where the samples were attached. The incident angle was selected as 50^o^, beyond the critical angle of silica glass *θ*_*c*_ = 43.2^o^. Far-field transmission spectra were collected from the flat surface of the prism. The light was collected by the integrating sphere and propagated to the spectrometer (Zolix Omni-300) via an optical fibre. A schematic diagram of the light-transmission beyond the critical angle measurement system is shown in Fig. 3d.

Preparation and characterisation of GaN-based LEDs. The GaN-based LED chips were provided by Shandong Inspur Huaguang Optoelectronics Company, Ltd., Jinan, China. Devices were grown on the c-plane of sapphire by metal-organic chemical vapour deposition (MOCVD). GaN-based LED chips were fabricated by sequentially growing undoped GaN (u-GaN, 2 *μ*m), n-GaN (2 *μ*m), five periods of InGaN/GaN multiple quantum wells (MQWs), p-GaN (250 nm), indium tin oxide (200 nm), and Cr/Au electrodes on c-plane sapphire substrates (400 *μ*m). PL measurements were carried out by exciting the LED sample from the bottom side of the sapphire substrate with a 405-nm laser diode and collecting PL signals both from the GaN side which has the Ag Meta-IV and sapphire substrate side. In the measurement of PL, Ag Meta-IV were directly deposited on top of the p-GaN layer without an ITO electrical contact layer. For EL measurement, the LED sample also consists of a 200-nm thick ITO layer evaporated onto the surface to serve as the upper electrical contact. The Meta–IV was deposited on top of the ITO electrical contact layer. Electrical and optical characteristics of the LED chips were measured using an on-wafer testing configuration (IPT 6000 LED chip/wafer probing system), comprising a parameter analyser and optical detectors mounted above the LED chips. Far-field radiation profiles of the LED chips were measured by using an LED goniophotometer (LED626, EVERFINE Corporation).

## Supplementary information

Supplemental Materials for Single-step-fabricated disordered metasurfaces for enhanced light extraction from LEDs
