# Supplementary material for: Single-step-fabricated disordered metasurfaces for enhanced light extraction from LEDs
Source: Light Sci Appl. 2021 Sep 6;10:180. doi: 10.1038/s41377-021-00621-7 (PMC8421350; doi:10.1038/s41377-021-00621-7)
Supplement: Supplementary file 1 — Supplemental Materials for Single-step-fabricated disordered metasurfaces for enhanced light extraction from LEDs [file 41377_2021_621_MOESM1_ESM.pdf]

Supplementary Information for "Single-step-fabricated disordered metasurfaces for enhanced light extraction from LEDs"

Peng Mao[1,2,†], Changxu Liu[1,3,†,*], Xiyan Li[4,†], Mengxia Liu[4,†], Qiang Chen[2,5], Min Han[2], Stefan A. Maier[3,6], Edward H. Sargent[4,*] & Shuang Zhang[1,7,8*]

[1]School of Physics and Astronomy, University of Birmingham, B15 2TT, United Kingdom,
[2]National Laboratory of Solid State Microstructures, College of Engineering and Applied Sciences and Collaborative Innovation Centre of Advanced Microstructures, Nanjing University, Nanjing210093, China
[3]Chair in Hybrid Nanosystems, Nanoinstitute Munich, Faculty of Physics, Ludwig-Maximilians-Universitaet Muenchen, 80539 Muenchen, Germany
[4]Department of Electrical and Computer Engineering, University of Toronto, 35 St. George Street,Toronto, ON M5S 1A4, Canada
[5]Key Laboratory of Intelligent Optical Sensing and Manipulation (Nanjing University), Ministry of Education, China
[6]Department of Physics, Imperial College London, London SW7 2AZ, UK
[7]Department of Physics, University of Hong Kong, Hong Kong, 999077 China
[8]Department of Electrical and Electronic Engineering, University of Hong Kong, Hong Kong, 999077 China

[*]To whom correspondence should be addressed: changxu.liu@physik.uni-muenchen.de, ted.sargent@utoronto.ca, s.zhang@bham.ac.uk

Supplementary Note 1: A comparison of state-of-art light-extraction structures

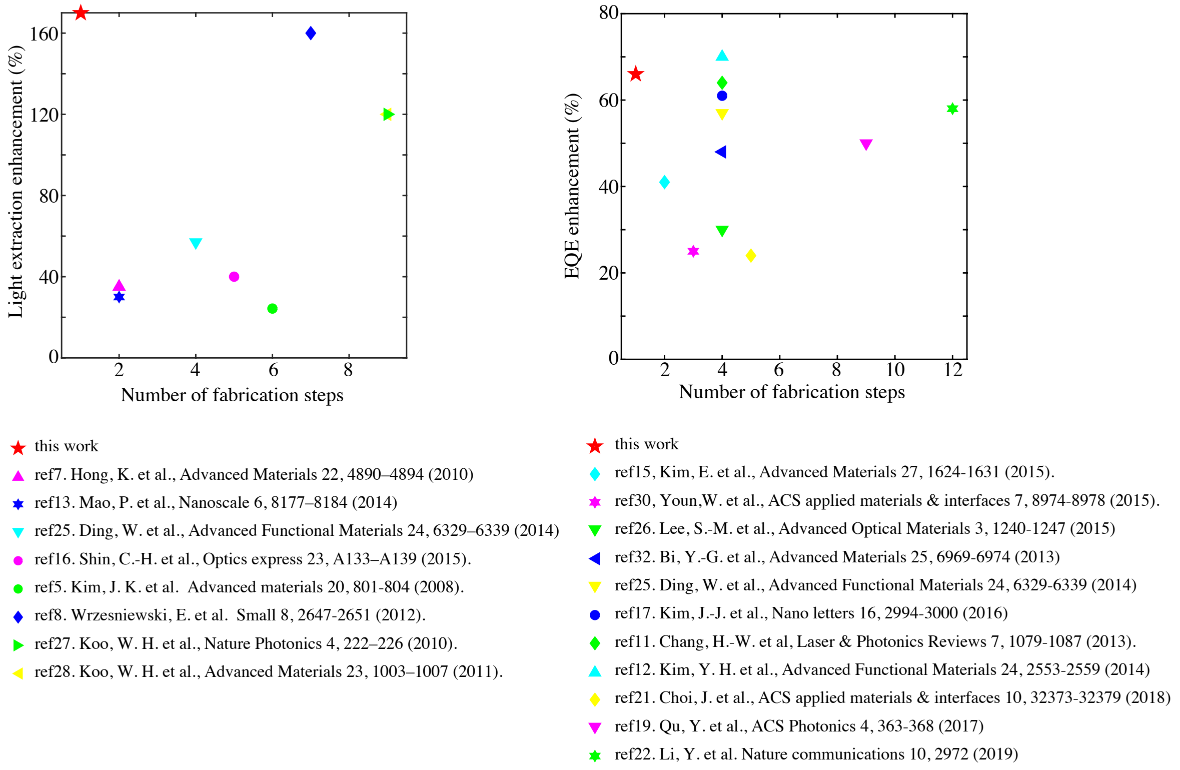

Supplementary Figure 1. A comparison between our work and state-of-art light-extraction structures. (a) Relationship between number of fabrication steps and enhancement of light extraction efficiency. (b) Relationship between number of fabrication steps and enhancement of EQE.

In this section, we implement a comparison between our metasurface and other state-of-the-art light extraction structures. Supplementary Figure 1 demonstrates the the performance improvment as a function of number of fabrication steps. The reference numbers correspond to the ones show in the main text. Supplementary Figure 1a illustrates the enhancement of light extraction while Supplementary Figure 1b shows the enhancement of external quantum efficiency (EQE). Simple fabrication with maximised enhancement is desired for practical applications. However, a trade-off between fabrication complexity and enhancement exists, as shown in Supplementary Figure 1. Our disordered metasurfaces provides a promising solution that achieves single-step fabrication and premier enhancement simultaneously. The absolute EQE is also provided Supplementary Figure 2 as a reference.

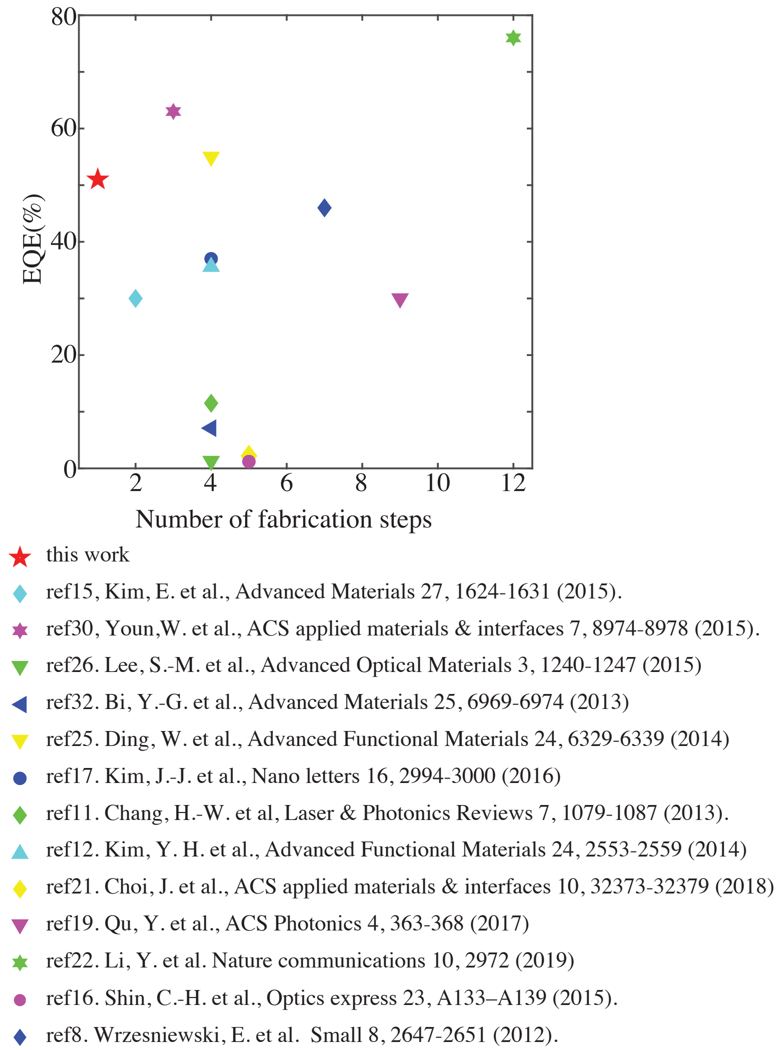

Supplementary Figure 2. A comparison of absolute EQE.

The fabrication process for devices listed in Supplementary Figure 1 are summarised below, providing a detailed picture of the fabrication complexity.

- this work
  Structure: Disordered Metasurface
  Fabrication steps:
  1. Gas-phase cluster beam deposition.
- ref5. Kim, J. K. et al. Advanced materials 20, 801-804 (2008)
  Structure: Graded-Refractive Index Indium Tin Oxide Anti-Reflection Contact

Fabrication steps:

1. Oblique-angle deposition at angle of 0°
2. Oblique-angle deposition at angle of -45°
3. Oblique-angle deposition at angle of 60°
4. Oblique-angle deposition at angle of -75°
5. Oblique-angle deposition at angle of 80°
6. Oblique-angle deposition at angle of -85°

- ref7. Hong, K. et al., Advanced Materials 22, 4890-4894 (2010)
  Structure: Nanofacet-structured refractive-index-modulating
  Fabrication steps:

  1. Deposition of MgO film.
  2. Deposition of ZrO_2 film.

- ref8. Wrzesniewski, E. et al. Small 8, 2647-2651 (2012)
  Structure: Molded Transparent Polymer Microlens Arrays
  Fabrication steps:

  1. PS monolayer-(2) -(3).
  2. Prepare PDMS.
  3. Thermal curing of PDMS.
  4. Remove PS
  5. Prepare NOA
  6. Place substrate
  7. Remove PDMS mold

- ref11. Chang, H.-W. et al, Laser & Photonics Reviews 7, 1079-1087 (2013)
  Structure: Nanoparticle-based scattering layer
  Fabrication steps:

  1. A homogenous dispersion is achieved by physical vibration, stirring, and milling the mixture for 24 h.
  2. The dispersion is subsequently filtered (pore size, 5μm) to remove the milling particles.
  3. Spin coating.
  4. Heat curing.

- ref12. Kim, Y. H. et al., Advanced Functional Materials 24, 2553-2559 (2014)
  Structures: Metal oxide-based light extraction systems
  Fabrication steps:
  1. Sputtering Sn.
  2. Annealing in vacuum.
  3. Annealing in air.
  4. Spin-coating planarization layer
- ref13. Mao, P. et al., Nanoscale 6, 8177?8184 (2014)
  Structures: Porous high refractive index nanoparticle films
  Fabrication steps:
  1. Nanoparticle beam deposition
  2. Oxidation in O_2
- ref15, Kim, E. et al., Advanced Materials 27, 1624-1631 (2015)
  Structures:
  Fabrication steps:
  1. Industrial-grade PEN (I-PEN) substrates
  2. Annealed for imidization
- ref16. Shin, C.-H. et al., Optics express 23, A133?A139 (2015)
  Structures: Nanoparticle scattering layer
  Fabrication steps:
  1. Polymer matrix 80°C for 1h.
  2. Two solutions mixing.
  3. Scattering later solution Ultra-sonic 15min.
  4. Spin coating.
  5. Annealing 65°Cfor 1h.
- ref17. Kim, J.-J. et al., Nano letters 16, 2994-3000 (2016)
  Structures: Biologically Inspired structure
  Fabrication steps:
  1. Photolithographic definition of thermoset and thermoplastic resist with precise alignment, and thermal reflow at a temperature higher than the glass transition temperature of the thermoplastic resin.
  2. Oxygen plasma treatment on stretched PDMS mold after PDMS replication.

3. Releasing the oxidized PDMS mold.
4. Replica molding with UV curable optical resin, and release of the hierarchical structures from the PDMS mold.

- ref19. Qu, Y. et al., ACS Photonics 4, 363-368 (2017)
  Structures:
  Fabrication steps:

  1. Deposition of SiO2 film.
  2. Photoresist was subsequently coated.
  3. The pattern was photolithographically defined.
  4. Etch of the SiO2 film
  5. The photoresist was removed by exposure to oxygen plasma.
  6. Deposition of Ag film.
  7. Deposition of Au film.
  8. Cold-weld bonding.
  9. The bonded glass slabs are diced into 1 by 1 in. squares, which were soaked in Remover PG to dissolve the sacrificial LOR layer to leave the metallic-coated grid.

- ref21. Choi, J. et al., ACS applied materials & interfaces 10, 32373-32379 (2018)
  Structures: Light-Scattering Layer of Dewetted Ag Nanoparticles
  Fabrication steps:

  1. Preparation of flexible substrate.
  2. PEDOT: PSS buffer layer coating.
  3. Ag deposition.
  4. Low temperature annealing.
  5. SU-8 Planarization

- ref22. Li, Y. et al. Nature communications 10, 2972 (2019)
  Structures: Tailor-made nanostructures
  Fabrication steps:

  1. The base and curing agent is mixed mechanically and then degassed in vacuum for 10 min.
  2. The mixture is then spin coated on pre-cleaned glass substrates at 1000 rpm for 1 min.

3. PDMS coated substrates are cured in an oven at varied temperatures for different heating time.
4. Substrates with PDMS are transferred to the RIE instrument.
5. After RIE treatment, samples are taken out to ambient environment with a humidity of 55% at room temperature.
6. The RIE-treated PDMS samples are vapor modified by FDTS for 24 h in a closed container in a glovebox.
7. After mixing and degassing, the PDMS mixture is carefully poured onto FDTS treated samples and then annealed in an oven at 80^oC for 1 hour.
8. Diluted NOA 63 by mixing with acetone at a weight ratio of 1:1 is spin coated on cleaned glass substrates.
9. The PDMS stamp is pressed into NOA 63 film by a home-made nano-imprinter and cured under UV radiation for 10 min.
10. Substrates with nanoimprinted NOA 63 is heated at 70^oC under vacuum for 5h before ITO sputtering.
11. The ITO films are grown by sheet-to-sheet processing in the pilot scale in-line sputter coater.
12. After sputtering, the ITO samples are annealed at 70^oC for 1 hour.

- ref25. Ding, W. et al., Advanced Functional Materials 24, 6329?6339 (2014)
  Structures: Plasmonic cavity with subwavelength hole-array
  Fabrication steps:

  1. Nanoimprint lithography.
  2. Deposition of Au film.
  3. Lift-off of 15 nm thick Au.
  4. An UV-ozone treatment to form an atomic thick AuO_x.

- ref26. Lee, S.-M. et al., Advanced Optical Materials 3, 1240-1247 (2015)
  Structures: Plasmonic Nanomesh Electrodes
  Fabrication steps:

  1. Close-packed PS particles were hexagonally arranged on a clean glass or polyethylene terephthalate (PET) substrate using the Langmuir?Blodgett method.
  2. Partial etching of PS particles by a RIE system.
  3. Deposition of Ag film.
  4. Elimination of PS particles.

- ref27. Koo, W. H. et al., Nature Photonics 4, 222-226 (2010).
  Structures: Buckling patterns
  Fabrication steps:
  1. Spin coating of PDMS.
  2. The coated PDMS was cured at 100°C for 1 h.
  3. An Al layer was deposited on the PDMS.
  4. After deposition of Al, the heated PDMS was cooled to ambient temperature by keeping the PDMS in the chamber for more than 30 min and venting to atmosphere.
  5. The PDMS replica was formed by pouring PDMS materials over the buckled PDMS master and by curing at 100°C for 1 h.
  6. The PDMS replica was peeled off the PDMS master.
  7. Second deposition of a 10-nm-thick Al layer on the buckled PDMS replica.
  8. The buckling pattern of the replica was transferred to a glass substrate using UV curable resin with UV curing for 10 min.
  9. The third deposition of a 10-nm-thick Al layer
- ref28. Koo, W. H. et al., Advanced Materials 23, 1003-1007 (2011).
  Structures: Buckling patterns
  Fabrication steps:
  1. Spin coating of PDMS.
  2. The coated PDMS was cured at 100°C for 1 h.
  3. An Al layer was deposited on the PDMS.
  4. After deposition of Al, the heated PDMS was cooled to ambient temperature by keeping the PDMS in the chamber for more than 30 min and venting to atmosphere.
  5. The PDMS replica was formed by pouring PDMS materials over the buckled PDMS master and by curing at 100°C for 1 h.
  6. The PDMS replica was peeled off the PDMS master.
  7. Second deposition of a 10-nm-thick Al layer on the buckled PDMS replica.
  8. The buckling pattern of the replica was transferred to a glass substrate using UV curable resin with UV curing for 10 min.
  9. The third deposition of a 10-nm-thick Al layer

- ref32. Bi, Y.-G. et al., Advanced Materials 25, 6969-6974 (2013).
  Structures: Corrugated Metallic Electrodes with Dual Periodicity
  Fabrication steps:

  1. A layer of photoresist was spin-coated onto the cleaned substrate.
  2. Exposed by two beams.
  3. Exposed for a second time after rotation by 60^oC
  4. Baked for 1 min (95^oC) and then developed in acetone.

Supplementary Note 2: Microstructures on the lantern cuticle of the fireflies

The firefly samples were treated under sonication to remove contaminant and further infiltrated with 2.3 mol/L sucrose overnight. The firefly samples were washed in distilled water to remove sucrose and then dehydrated through ethanol solutions of 50%, 70%, 90%, and 95% (vol/vol) twice for 10 min each and 100% three times for 10 min. Subsequently, the sample was treated with hexamethyldisilazane (HMDS; EMS) and air-dried overnight. To observe the microstructures of the sample by using SEM, the gold thin layer was coated on the sample surface using thermal evaporation method. The prepared sample were then attached to a copper SEM stub by using carbon tape. A high-resolution field-emission scanning electron microscope (HITACHI S4800) was used for analyzing the nanostructures of firefly lantern as shown in Supplementary Figure 3.

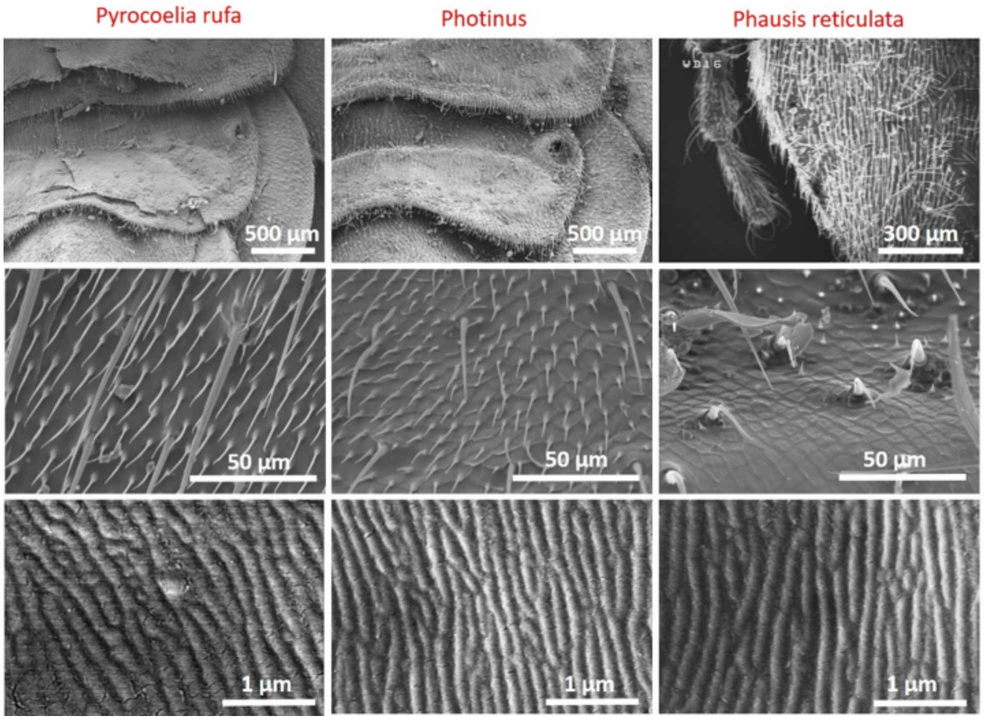

Supplementary Figure 3. SEM images of lantern cuticles of the three different types of fireflies.

Supplementary Note 3: Investigation of the impact of periodicity and particle size in light extraction

In the manuscript, the periodicity Λ is selected as 230nm (half of the wavelength blue light) and the particle size d is selected as 100 nm. To provide a comprehensive picture, we provide additional investigation to show how the periodicity and particle size impact the light extraction beyond the critical angle. For simplicity but without loss of generality, we investigate 2D periodic structures without disorder.

Figure S4 summarises the influence of the periodicity at different incident angles above the critical angle. With the decrease of the periodicity, a blue shit of the transmission spectra is observed. Meanwhile, additional region with enhanced transmission is observed (comparing Figure S4c and b). Similarly, Fig. S5 summarises the influence of the periodicity at different incident angles above the critical angle. A blue shift of the transmission spectra is observed as the particle size decreases, due to the scalability of Maxwell's equations. With the variation of the size, regions with enhanced photon extraction are introduced and shifted, similar to the case of varying periodicity. Due to the complexed relationship between light extraction and periodicity/size even in 2D periodic structures, we directly optimise the metasurfaces from experiments, as shown in Supplementary Note 6.

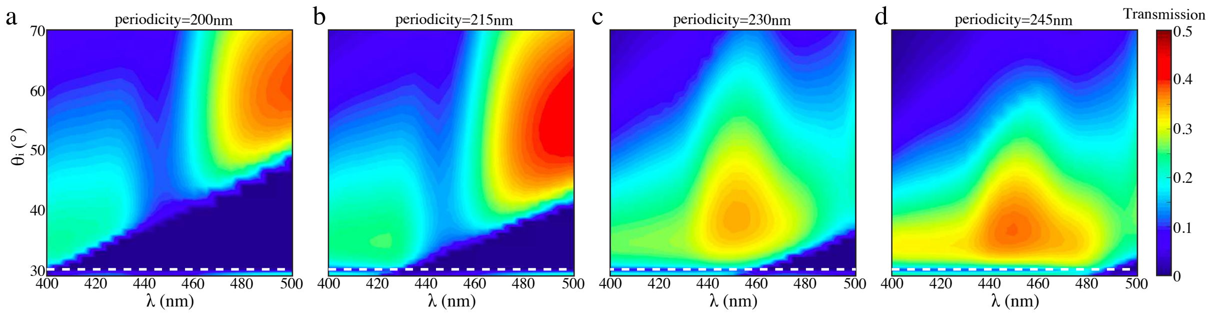

Supplementary Figure 4. The impact of periodicity on light extraction beyond critical angle. The particle size is fixed to 100nm. Structures with different periodicities are shown respectively: a) Λ=200nm; b) Λ=215nm; c) Λ=230nm; d) Λ=245nm.

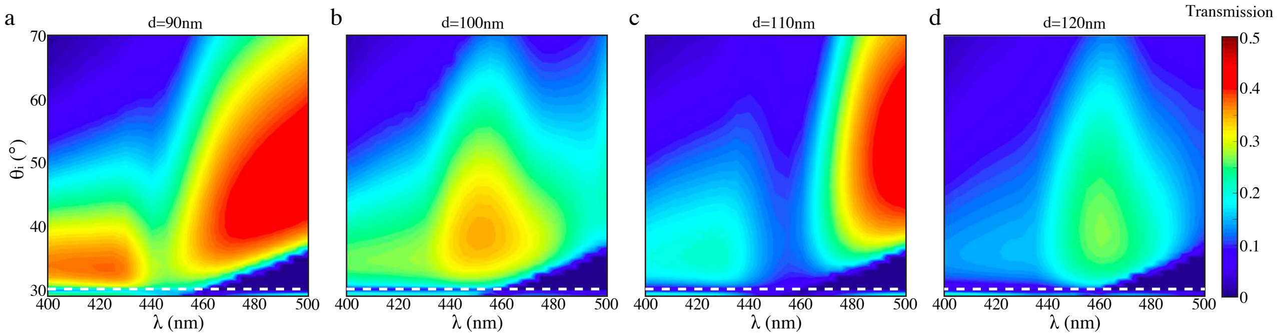

Supplementary Figure 5. The impact of particle size on light extraction beyond critical angle. The periodicity is fixed to 230nm. Structures with different particle sizes are shown respectively: a) d=90nm; b) d=100nm; c) d=110nm; d) d=120nm.

Supplementary Note 4: Investigation of the impact of disorder in light extraction

Here we investigate how the magnitude of the fluctuations in size (Δ_{siz}) and position (Δ_{pos}) influences the transmission T_{TIR} beyond total internal reflection. Transmission spectra at three different wavelengths are simulated, with the results summarised in Fig. S6. To clarify the effect of disorder, we demonstrate the variation between the disordered and ordered structure, $\Delta T = T_{\text{TIR}} - T_{\text{TIR},0}$. $T_{\text{TIR},0}$ is the transmission spectrum of ordered structure with the particle size of 100nm and a period of 230 nm. The incident angle is fixed to 32°. The results are averaged from three different random sets.

The disordered-induced transmission change ΔT has a strong dependence on the wavelength. While the disorder improves the transmission at 500nm (Fig. S6c), the enhancement at 400nm (Fig. S6a) or 450nm (Fig. S6b) is observed in specific regions with optimised values of Δ_{siz} and Δ_{pos}. Nevertheless, some regions with transmission enhancement (reddish-coloured area) coincide at three different wavelengths, illustrating a broadband improvement. In addition, the combination of disorder in position and size is desired for better transmission.

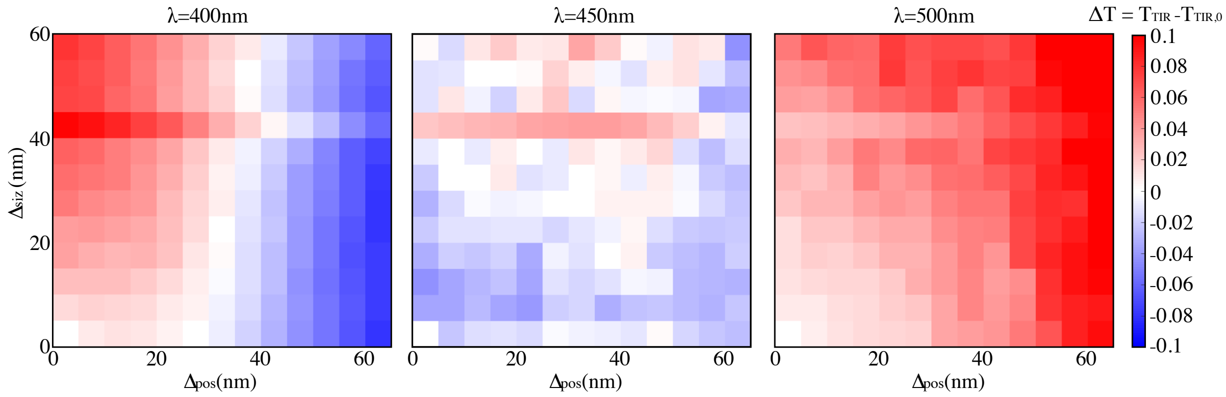

Supplementary Figure 6. The relationship between the magnitude of disorder and the variation of the transmission under total internal reflection at different wavelengths; (a)λ=400nm, (b)λ=450nm, (c)λ=500nm. Δ_{siz} is the fluctuation in the size and Δ_{pos} s the fluctuation in the position. ΔT is the variation of the transmission compared with ordered structure.

We also investigate the impact of the density of NPs on the light extraction, with the results summarised in Fig. S7. Here, the fluctuations of the size of NPs Δ_{siz} is also included. We fix the disorder in position to 10% of the average distance among the NPs. The results are averaged from three different random sets. The density plays an important role in light extraction. There are optimised densities for outcoupling the photons, as shown in Fig. S7a-c. Such effect also has a strong wavelength-dependence. In addition,

we observe a disorder enhanced transmission when magnitude of Δ_{siz} is moderate, similar to the results shown in Fig. S6.

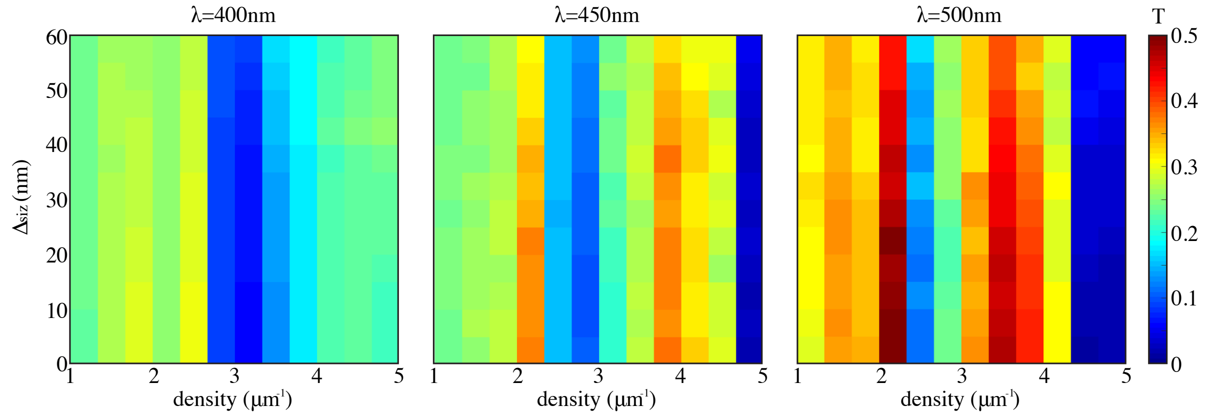

Supplementary Figure 7. The relationship between the NP density/disorder in size and the transmission under total internal reflection at different wavelengths; (a)λ=400nm, (b)λ=450nm, (c)λ=500nm.

Supplementary Note 5: Artificial design step for the disordered metasurface

In the main text, the simulations demonstrated the effect of light extraction based on the features from bio-inspiration. Here, the effect of structure extension is investigated, clarifying the pivotal of artificial design from Meta-III to Meta-V. Considering the limited computational resources, we investigate the periodic structures demonstrated in Supplementary Fig. 8a and b. The periodic stripes with a curved top surface are extended to a periodic structure composed of nanoparticles with a spherical surface on top. The effect of disorder is directly shown by the experimental results in the main text and Supplementary Note 5.

Supplementary Fig. 8c compares the transmission T at normal incidence when the polarisation of the electric field varies in the x-y plane with different azimuthal angle ϕ. The difference in transmission clearly demonstrates another benefit from structure extension - the reduced coverage rate facilitates more photons to go outside the substate inside the light cone (no TIR occurs). Supplementary Fig. 8d investigates the case when incident angle θ changes. The azimuthal angle ϕ is set to 0° when the stripe obtains maximum transmission. Even under this situation, the nanoparticle can extract more photons, owing to the enhanced capability for coupling light above the critical angle θ_c.

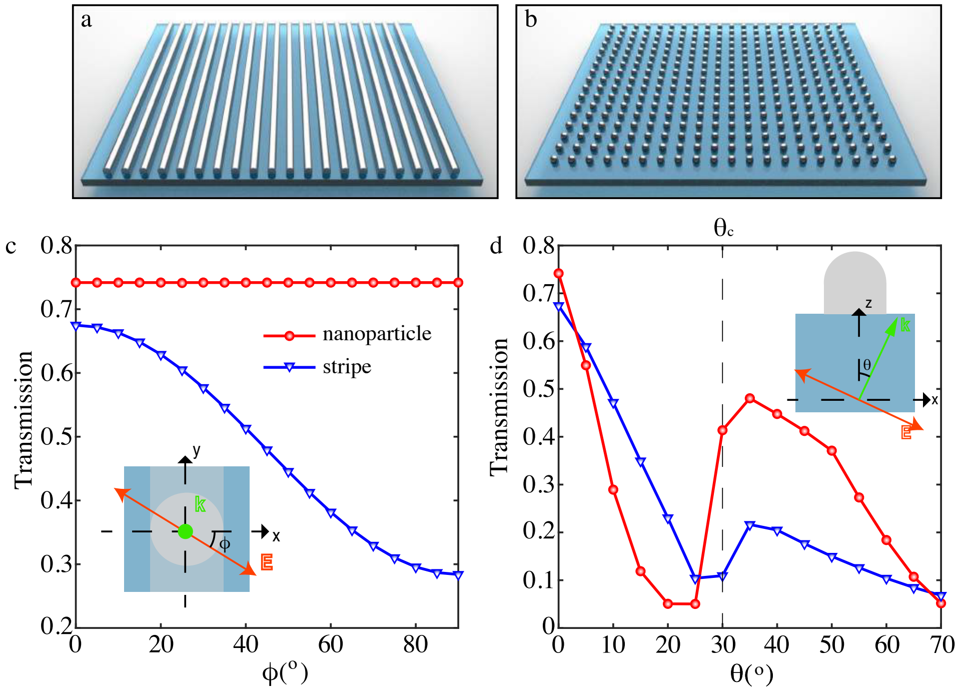

Supplementary Figure 8. (a) Metasurface composed with periodic stripes (b) Metasurface composed with periodic nanoparticles. (c) The transmission of the two types of metasurfaces as a function of azimuthal angle ϕ under normal incidence. The inset shows the azimuthal angle ϕ. (d) The transmission of the two types of metasurfaces as a function of incident angle θ. Azimuthal angle ϕ is fixed to 0° when the stripe obtains maximum transmission. The inset shows the incident angle θ.

Supplementary Note 6: Fabrication, characterisation and optimisation of disordered Ag metasurfaces

Ag metasurfaces were prepared by gas-phase nanocluster beam deposition method [2]. The nanocluster beam deposition system is composed of a cluster source, differential vacuum component, particle control component and a deposition chamber. In this fabrication, Ag clusters were generated in a magnetron plasma gas aggregation cluster source. The nanocluster beam was formed by differential pumping induced expansion, and then deposited onto the surface of the LED chips directly. The deposition was performed in a high-vacuum chamber equipped with the cluster source. An Ag target with high purity (99.999%) was used as the sputtering target. The magnetron discharge was operated in an argon stream at a pressure of about 90 Pa in a liquid nitrogen cooled aggregation tube. Ag atoms were sputtered out from the target and Ag nanoclusters were formed through the gas aggregation process in the argon gas. The clusters were swept by the gas stream into high vacuum through a nozzle and a skimmer, respectively, forming a collimated cluster beam with ultrasonic speed. Thus, the nanoparticles deposited and stuck on the substrate or LED chip surface firmly.

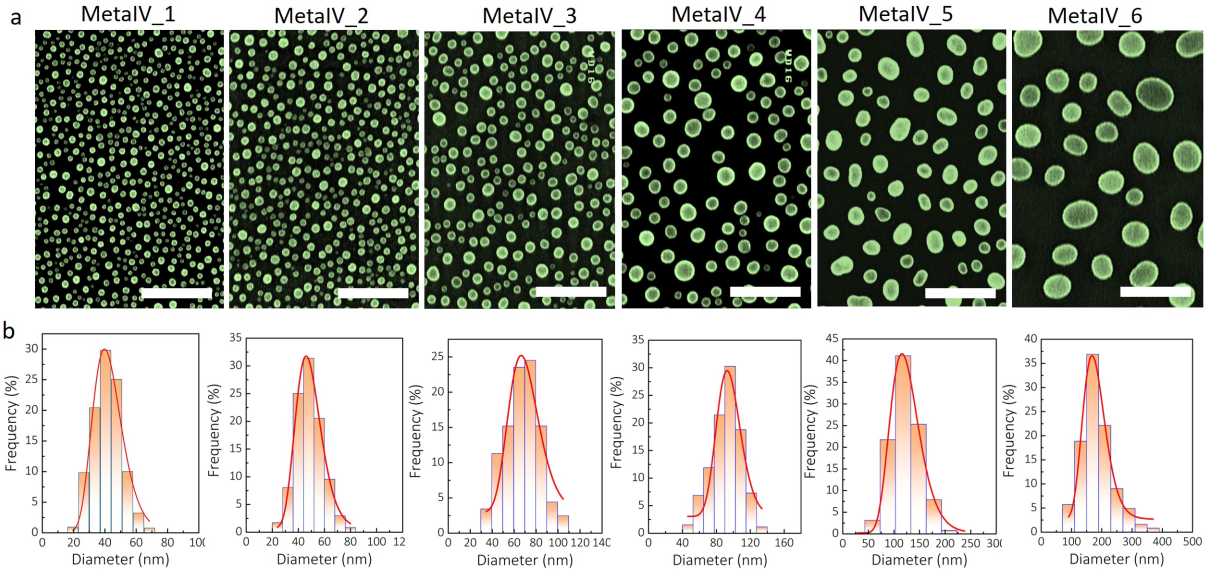

Supplementary Figure 9. (a) SEM images of disordered metasurfaces with different particle size. The scale bar represents 500nm. (b) Corresponding statistical analysis of the size of Ag nanoparticles in each metasurface. A Log-Normal fitting is also provided.

The gas-phase cluster beam technique provides the flexibility to form Ag nanoparticles with different sizes and coverage rate by simply tuning the deposition time and temperature. The deposition was carried out at a rate of 0.6 ± 0.1 Å s^{-1}. The deposition time is 5 min, 9min, 12 min, 15 min, 18 min and 21 min. The post-deposition

rapid annealing process was performed for 25 min in situ with a temperature of 400°C. The structural properties of the metasurfaces were characterised by scanning electron microscopy (SEM, HITACHI S4800), as shown in Supplementary Fig. 9a. Due to the fact that silver nanoparticles have high mobilities on the surface of substrates[1], diffusion and coalescence of metallic nanoparticles on sapphire surface induce a significant increase in nanoparticle size with the deposition mass. The corresponding size distribution histograms of the deposited clusters are given in Supplementary Fig. 9b, which show that averaged size of Ag nanoparticles covers a range from 40 nm to 185 nm. Diameters of the deposited clusters observed from SEM are much larger than those of the clusters in the free beam, indicating that the clusters are effectively coalesced after they land on the surface.

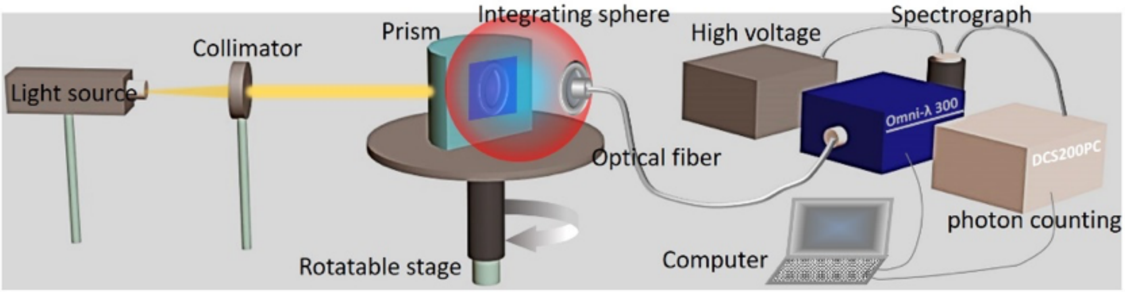

Supplementary Figure 10. Schematic diagram of the light extraction measurement system

We perform transmission measurement for each metasurface with the setup shown in Supplementary Fig. 10. The results are summarised in Supplementary Fig. 11. From the transmission spectra (Supplementary Fig. 11a), the Meta-IV_6 is selected as the optimised structure for the light extraction. The performance of the metasurface can be evaluated from naked eyes through the images in Supplementary Fig. 11b. The transmitted light from Meta-IV_6 not only has a stronger intensity but also processes a white colour, implying a broadband light extraction.

We further increase the size of the NPs, with the results demonstrated in Fig. S12. An apparent degradation in light extraction is observed for the metasurface with an averaged NP size of 231nm, demonstrating the Meta-IV6 be the best one available for light extraction. NPs above 231nm can be fabricated at a high temperature. But the temperature may impair the performance of LEDs beneath the metasurfaces.

Due to the presence of several degrees of freedom in fabrication, there may be additional space to improve the extraction efficiency by seeking an optimised recipe. The recent development of deep-learning-aided recipe optimisation [3] may be utilised in future works for even better performance.

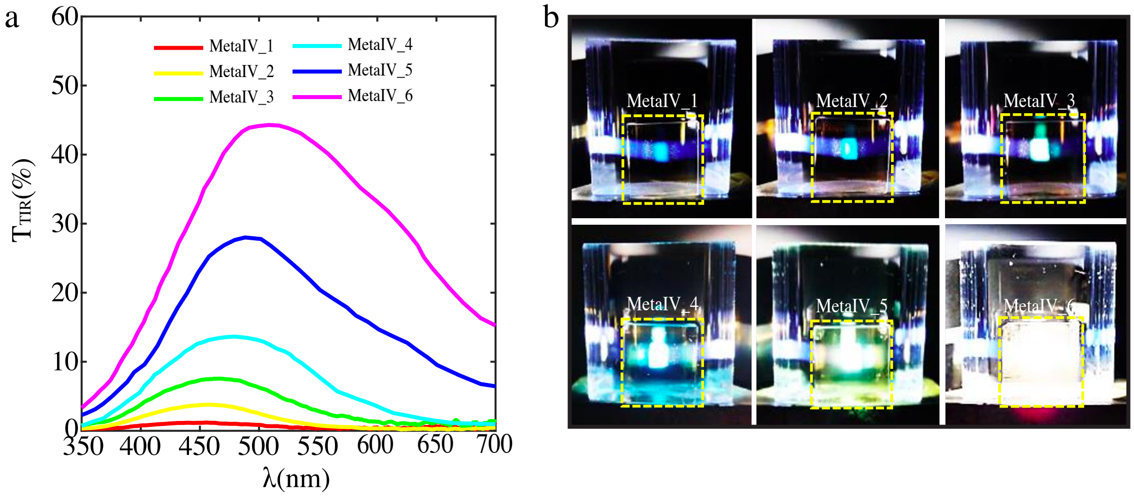

Supplementary Figure 11. (a) Transmission spectra for different metasurfaces under TIR. (b) Corresponding optical photographs of the transmitted light using different metasurfaces.

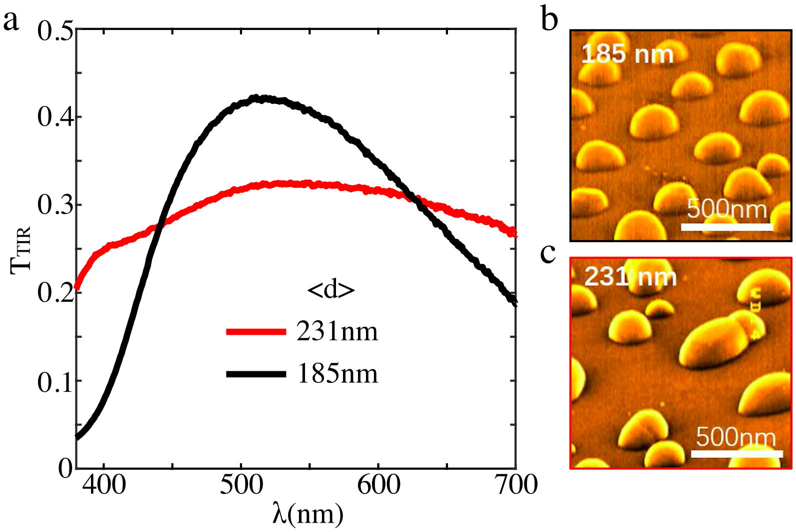

Supplementary Figure 12. (a) Transmission spectra for metasurfaces with large NP size. (b-c) Corresponding SEM images of the two metasurfaces.

Supplementary Note 7: Additional 3D simulations based on the experimental samples

Full-wave 3D simulations are implemented for Meta-IV, as shown in Fig. SFig. S13. The parameter of nanoparticles is based on the configuration from the experiments. The diameter of hemisphere d obeys a Gaussian distribution with a mean of 185 nm and a deviation of 45nm. The height of the cylinder below the hemisphere is half of the radius. The period is 400 nm and Δ_{pos} is equal to 100nm. The results are averaged with four different random sets.

The maximum transmission occurs around 600nm in the simulation, while the transmission peak was experimentally observed around 500nm. We attribute this blue shift to the deformation of the top half of real nanoparticles (compared to a hemisphere), as shown in Fig. 2c in the main text. We attribute the 100nm blue shift in the transmission spectrum to the volume reduction in real nanoparticles. Importantly, broadband transmission above the critical angle is demonstrated in both simulation and experiment. The maximum value of T_{TIR} from experiments is larger than the simulated one, due to the shape variation of nanoparticles in fabrication. The moderate increment of the disorder resulted from fabrication imperfection can further enhance the transmission, matching the results shown in the main text and Supplementary Note 4.

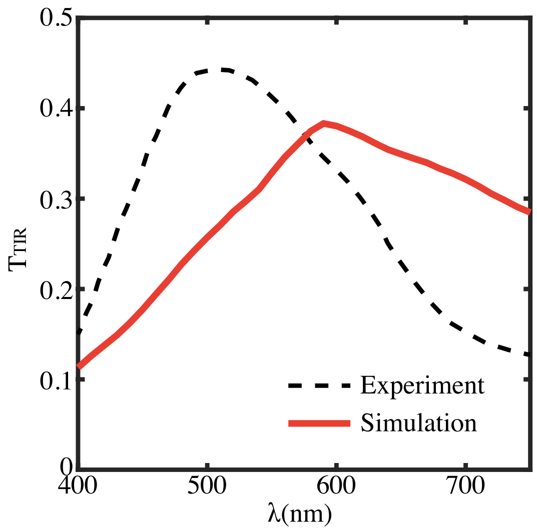

Supplementary Figure 13. 3D Simulations for Meta-IV. The transmission T_{TIR} is calculated with incident angle of 50°. Experimental results are also shown as a reference. The blue shift of the experimental spectrum is due to the deformation of the top half of fabricated nanoparticle.

Supplementary Note 8: Angle dependence of the light extraction from Meta-IV

The angle dependence of the light extraction is investigated experimentally, with results summarised in Fig. S14 and Fig. S15. Figure S14a illustrates the setup. An integrating sphere is utilised to collect all the scattered photons. The transmission spectra are shown in Fig. S14b, with incident angles ranging from 50° to 68°. In spite of the decrease in transmission at larger incidence angles, photons in a broad spectral range can be extracted by utilising the disordered metasurface.

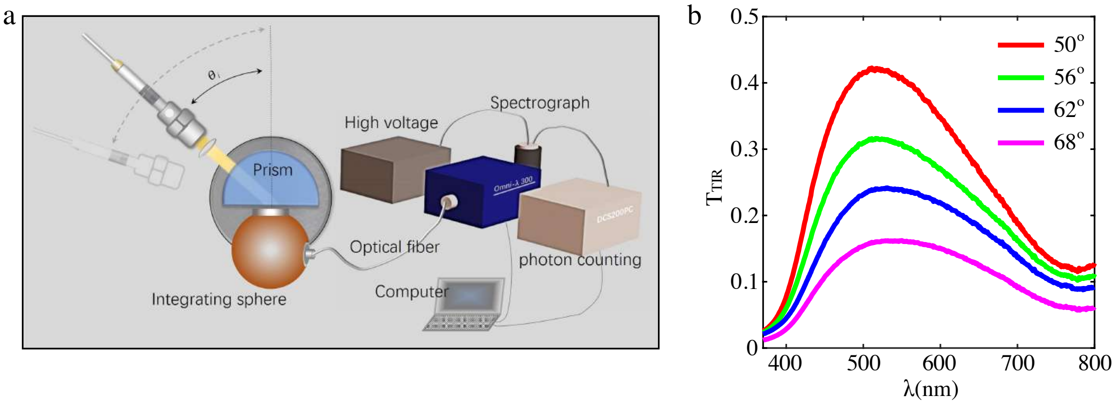

Supplementary Figure 14. Angle dependence of the transmission beyond critical angle from Meta-IV. (a) The experimental setup. (b) Transmission spectra T_{TIR} for the incident angles beyond the total internal reflection.

Figure S15 summarises the light extraction below and above the critical angle at a fixed wavelength of 473nm. The transmission of a configuration without metasurface is also provided as a reference. Figure S15a and b show the photos for the setup with and without Meta-IV ,respectively. Figure S15c illustrates the relationship between the incident angle and transmission. Below the critical angle, a portion of photons is blocked by the nanostructure on top compared to the reference. But overall, the metasurface facilitates the light extraction, regarding the significant improvement of the transmission above the critical angle. This light extraction ability is also confirmed by the improvement of the EQE of LEDs.

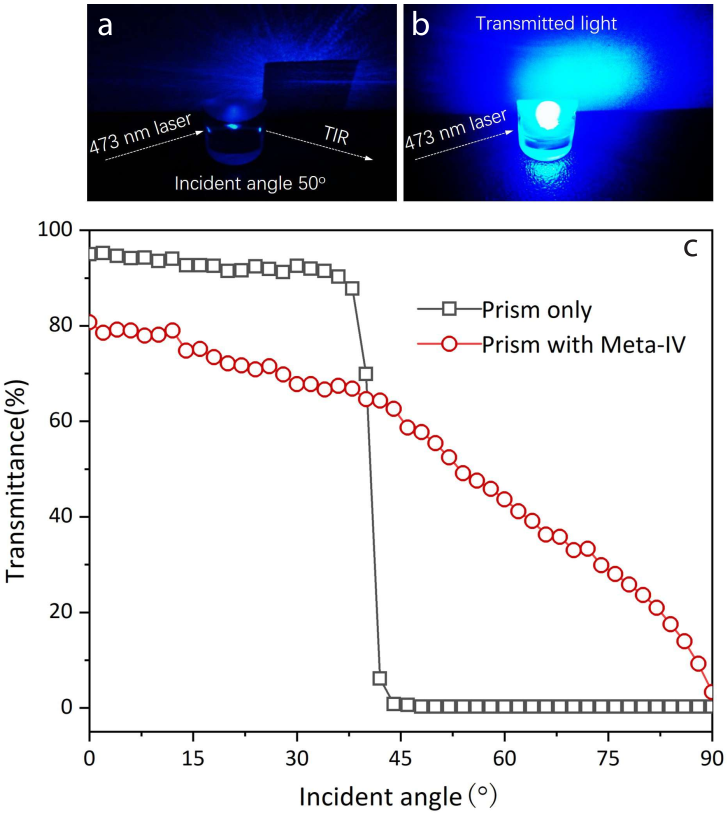

Supplementary Figure 15. Angle dependence of the transmission at a fixed wavelegth. (a-b) Photos for the setups with and without Meta-IV at the incident angle of 50°. A huge enhancement of light extraction is observed. (c) The relationship between Transmission T and incident angle.

Supplementary Note 9: PL measurement at different input powers

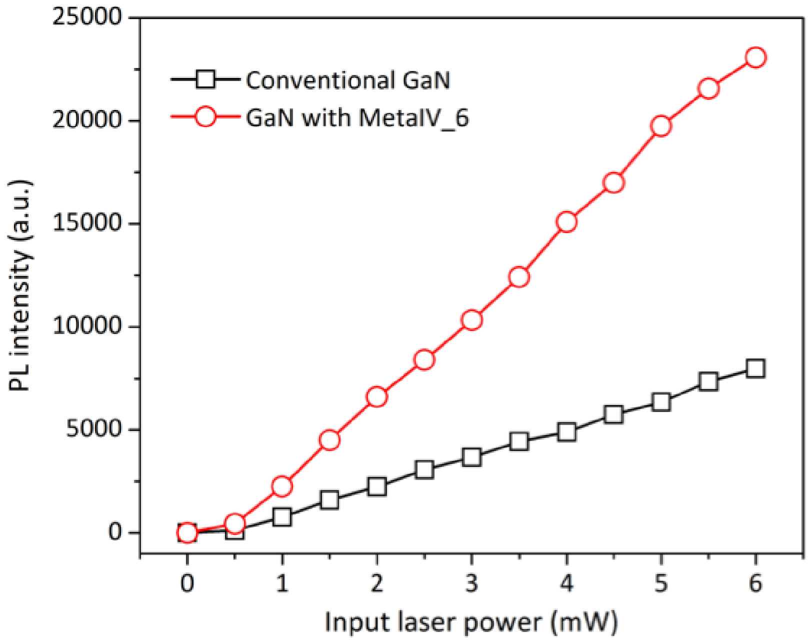

Supplementary Figure 16. Room temperature PL intensity at 450nm wavelength of two samples (bare GaN LEDs, GaN LEDs with MetaIV_6 structure) under various excitation laser powers.

Supplementary Note 10: Internal quantum efficiency (IQE) as a function of excitation power

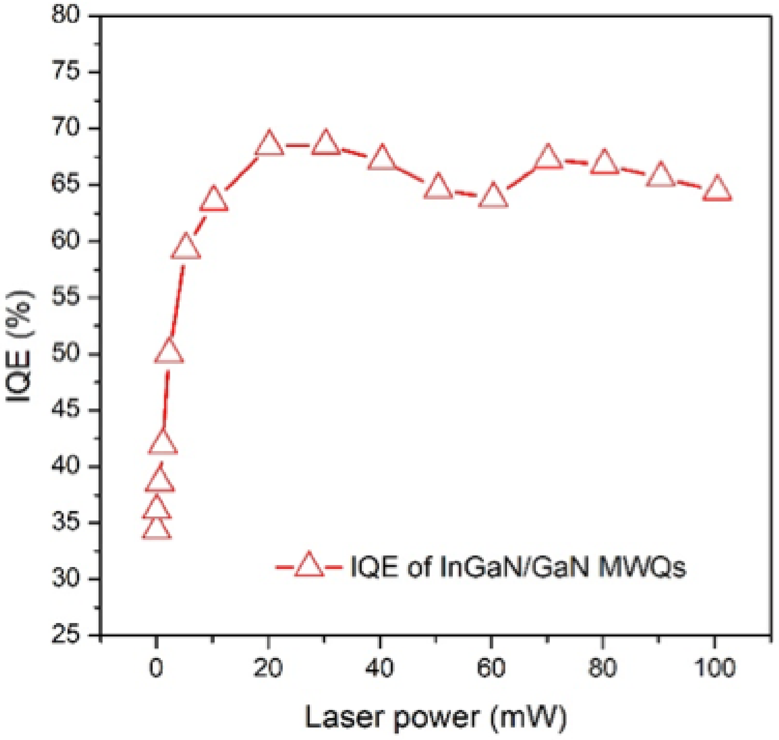

Supplementary Figure 17. Internal quantum efficiency of InGaN/GaN multiple quantum well (MQW) as a function of excitation power.

Supplementary Note 11: Additional information for EQE measurement

After dicing and splitting, the LEDs were made into independent chips, and fixed with silver glue in the reflective bowl. Then the chips were connected with gold wires and covered the semi-circular epoxy resin filled it with silicone resin, and heat-cure to obtain the encapsulated LEDs.

The relationship between light output power (LOP) and current(I) of encapsulated blank LED and encapsulated LED with Meta VI was measured and shown in Supplementary Fig. 18.

The external quantum efficiency (EQE) of LED can be calculated through

$$EQE = \frac{LOP}{IV} \tag{1}$$

The measurement was implemented with 75 independent chips, with statistical results demonstrated in Supplementary Fig. 19 - 20. Supplementary Fig. 19 shows the statistics of LOP while Supplementary Fig. 20 shows the statistics of EQE. An EQE enhancement from 31.6($\pm$ 2.6)% to 51.5($\pm$ 3.8)% is observed by virtue of the light extraction structure.

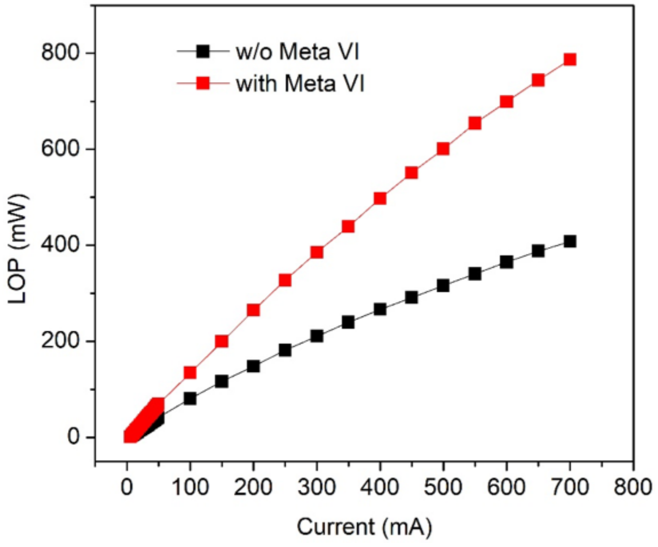

Supplementary Figure 18. LOP-I curves of encapsulated blank LED and encapsulated LED with Meta VI.

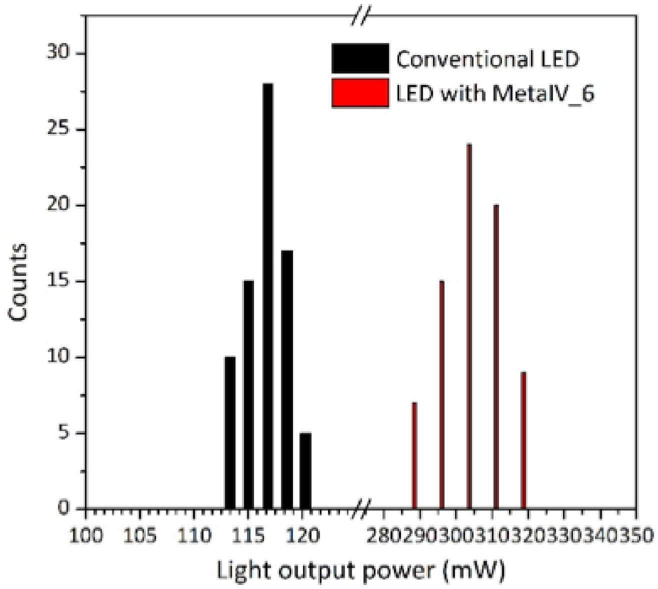

Supplementary Figure 19. Histograms of light-output power (LOP) measured from a set of two kinds of LEDs (conventional LED and LED with MetaIV_6), with each kind of LED composed of 75 separate chips.

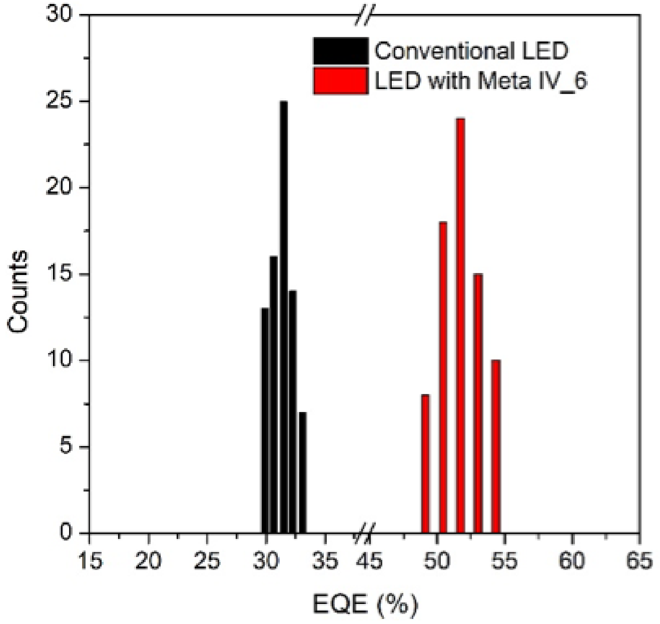

Supplementary Figure 20. Histograms of EQE measured from a set of two kinds of LEDs (conventional LED and LED with MetaIV_6), with each kind of LED composed of 75 separate chips.

Supplementary References

[1] C Binns. Nanoclusters deposited on surfaces. *Surface Science Reports*, 44(1-2):1–49, 2001.

[2] Peng Mao, Changxu Liu, Qiang Chen, Min Han, Stefan A Maier, and Shuang Zhang. Broadband sers detection with disordered plasmonic hybrid aggregates. *Nanoscale*, 12(1):93–102, 2020.

[3] Benjamin J Shields, Jason Stevens, Jun Li, Marvin Parasram, Farhan Damani, Jesus I Martinez Alvarado, Jacob M Janey, Ryan P Adams, and Abigail G Doyle. Bayesian reaction optimization as a tool for chemical synthesis. *Nature*, 590(7844):89–96, 2021.
